# Association between single nucleotide polymorphism of rs1937 in TFAM gene and longevity among the elderly Chinese population: based on the CLHLS study

**DOI:** 10.1186/s12877-021-02655-3

**Published:** 2022-01-03

**Authors:** Qing Chen, Zhi-Hao Li, Wei-Qi Song, Yao Yao, Yu-Jie Zhang, Wen-Fang Zhong, Pei-Dong Zhang, Dan Liu, Xi-Ru Zhang, Qing-Mei Huang, Xiao-Yang Zhao, Xiao-Ming Shi, Chen Mao

**Affiliations:** 1 Department of Developmental Biology, School of Basic Medical Sciences, Southern Medical University, Guangzhou, China; 2 Department of Epidemiology, School of Public Health, Southern Medical University, Guangzhou, China; 3 Center for Healthy Aging and Development Studies, National School of Development, Peking University, Beijing, China; 4 State Key Laboratory of Organ Failure Research, Department of Developmental Biology, School of Basic Medical Sciences, Southern Medical University, Guangzhou, China; 5 Guangdong Provincial Key Laboratory of Construction and Detection in Tissue Engineering, Southern Medical University, Guangzhou, China; 6 National Institute of Environmental Health, Chinese Center for Disease Control and Prevention, Beijing, China

**Keywords:** Longevity, TFAM, rs1937, Single nucleotide polymorphism, Long-lived individuals

## Abstract

**Background:**

To investigate whether the mitochondrial transcription factor A (TFAM) rs1937 single nucleotide polymorphism (SNP) is associated with longevity.

**Methods:**

We conducted a case-control study among Chinese long-lived individuals (≥90 years). Data were obtained on 3294 participants who were able to voluntarily provided a saliva sample during 2008–2009 from the Chinese Longitudinal Healthy Longevity Survey (CLHLS). In this study, 1387 young elderly (65–74 years) were allocated to the control group, and 1907 long-lived individuals were recruited as the case group. SNP rs1937 on TFAM were genotyped. Logistic regression models were applied to evaluate the association between rs1937 SNP and longevity.

**Results:**

The genotype frequency of the SNP of rs1937 in the two groups had a significant difference (*p* = 0.003). Binary logistic regression analysis showed that compared to younger elderly, the long-lived individuals with “CC genotype” of rs1937 were more closely related to increased longevity than those with “GG genotype” (OR: 1.989, 95% *CI*: 1.160–3.411). The positive association between rs1937 SNP and longevity was robust in stratified analyses and sensitivity analyses.

**Conclusions:**

We found the SNP of rs1937 may be a potential biomarker for longer human life span. Further studies are necessary to elucidate the biological mechanism of rs1937 on TFAM with promoting longevity.

**Supplementary Information:**

The online version contains supplementary material available at 10.1186/s12877-021-02655-3.

## Background

Mitochondria are one of the most important and intense studied organelles involved in cellular senescence and organismal aging [1], and they contain mitochondrial DNA (mtDNA) of the extranuclear genome. Mitochondria also have been known as the major source and target of reactive oxygen species (ROS) [2–4] and the main sites of adenosine triphosphate (ATP) production [5, 6]. The ROS produced by mitochondria can cause oxidative damage to mtDNA, thereby inducing mtDNA mutations, blocking the defective electron transport chain and generating more ROS, which can lead to cell damage and accelerate the body’s rate of aging. Previous researches have shown that chronic mtDNA damage and dysfunction are one of the main features for neurodegenerative diseases [7–10], human aging [9, 11, 12], as well as other age-related diseases [13–15].

Located on 10q21.1 and encoded by mitochondrial nuclear genes, mitochondrial transcription factor A (TFAM) has been found to be an essential regulatory factor in mtDNA initiation, transcription, and replication [16, 17]. Relevant researches have revealed that an appropriate increased level in the protein of TFAM can reverse neuron damage and may be involved in the prevention and control of ageing and neurodegeneration [13, 18, 19]. Meanwhile, several population-based studies [13, 19–21] have suggested that the single nucleotide polymorphism (SNP) of rs1937 on TFAM were associated with Alzheimer’s disease (AD). A recent study also investigated TFAM deficiency could induce multimorbidity and premature senescence mediating by T cells with dysfunctional mitochondria [22]. However, to the best of our knowledge, only one study [23] reported a possible marginal association of rs1937 with extreme longevity in Spain. Therefore, relatively little is known about the associations between SNPs rs1937 and human longevity, particularly in the Chinese long-living individuals.

To address this knowledge gap, using data obtained from the Chinese Longitudinal Healthy Longevity Survey (CLHLS) study, we conducted a case-control study on examining the association of SNP rs1937 with longevity among the Chinese old adults.

## Methods

### Study setting and participants

Data for the present study were obtained from the 5th wave of the CLHLS in 2008–2009. The CLHLS is a large population-based cohort study conducted in 23 provinces, municipalities, and autonomous regions. The populations together of this survey constitute approximately 85% of the total population in China. Previous studies have described the survey design and participants of CLHLS in detail [24–26]. Genetic samples and data collected in the CLHLS have used internationally standardized questionnaires adapted to the Chinese social and cultural environment, and have been successfully applied to the genetic studies [25–28].

In the present study, the inclusion criteria of the long-living individuals group was that those who were 90 years or older; the inclusion criteria of younger elderly group was that those who were in the age range of 65–74 years, and had no family history of longevity (no family member aged ≥90 years for at least two generations). All participants in two groups accepted to participate in the questionnaire survey and voluntarily provided a saliva sample. Participants who met one of the following criteria were excluded: (1) had incomplete baseline key variables; (2) declined to provide a saliva sample or take the genotyping test of TFAM; (3) aged 61-64 or 75–89 years. The final sample included 3294 participants: 1387 younger elderly (aged 65–74 years) as the controls, 1907 nonagenarians (aged 90–99 years), and centenarians (aged 100 years or older) as the cases. We found the distribution of the variables were similar whether non-Han participants were included or not (Additional file 1: Table S1). The CLHLS was approved by the Research Ethics Committee of Peking University (IRB00001052–13074). No experimental interventions were performed. All participants or their legal proxy signed written informed consent forms before participation.

### Ascertainment of longevity

Longevity is one of the commonly used medical terms for aging research that can be defined as the ability of the human to survive beyond the average age of death and reach a considerably longer life span under optimal conditions [29–31]. Following a previous study [32], the elderly with physiological aged 90 years and older are called long-lived individuals. In the present study, the long-lived population was defined as the observation index of longevity.

### Covariates

Data on the potential confounders were collected via the CLHLS questionnaire and classified as follows: sociodemographic information, including age (years), gender (men or women), living alone status, education (literacy, receiving a formal education ≥1 year; illiteracy, receiving a formal education<1 year), and body mass index (BMI, underweight < 18.5 kg/m^2^; normal weight, 18.5–23.9 kg/m^2^; overweight or obesity, ≥ 24.0 kg/m^2^) [33]; lifestyle behaviors, including the status of current smoking, current drinking, and multimorbidity. In this study, “current smoking” refers to those elderly who continue to smoke at least an average of one cigarette per day for more than a year, and “current drinking” refers to those elderly who continue to drink at least an average of one or two alcoholic drinks per day for more than a year; “multimorbidity” refers to those elderly who suffer from the co-existence of two or more chronic diseases [34, 35], such as hypertension, diabetes mellitus, respiratory disease, stroke, or heart disease.

### Genotyping and imputation

Genotyping was performed on Illumina Human OmniZhonghua-8 Beadchips, which represented a state-of-the-art choice for genome-wide association study (GWAS) in Asian populations to maximize international compatibility. It included 600 K SNPs of common variations (minor-allele frequency [MAF] ≥ 0.05), 290 K SNPs of rare variations (MAF < 0.05) as well as 10 K SNPs existing just in Chinese and other Asian populations.

Initial data quality control of the genotyped samples was performed by PLINK 1.06. Meanwhile, imputation analyzes, applying for IMPUTE software version 2 and the 1000 Genomes Project (1 KGP) integrated phase 1 release as reference panel, were conducted to infer the SNPs genotyping of rs1937 on TFAM (MAF ≥ 0.01). SNP of rs1937 with a quality score of < 0.9 was dropped before analyzes.

**Fig. 1:**
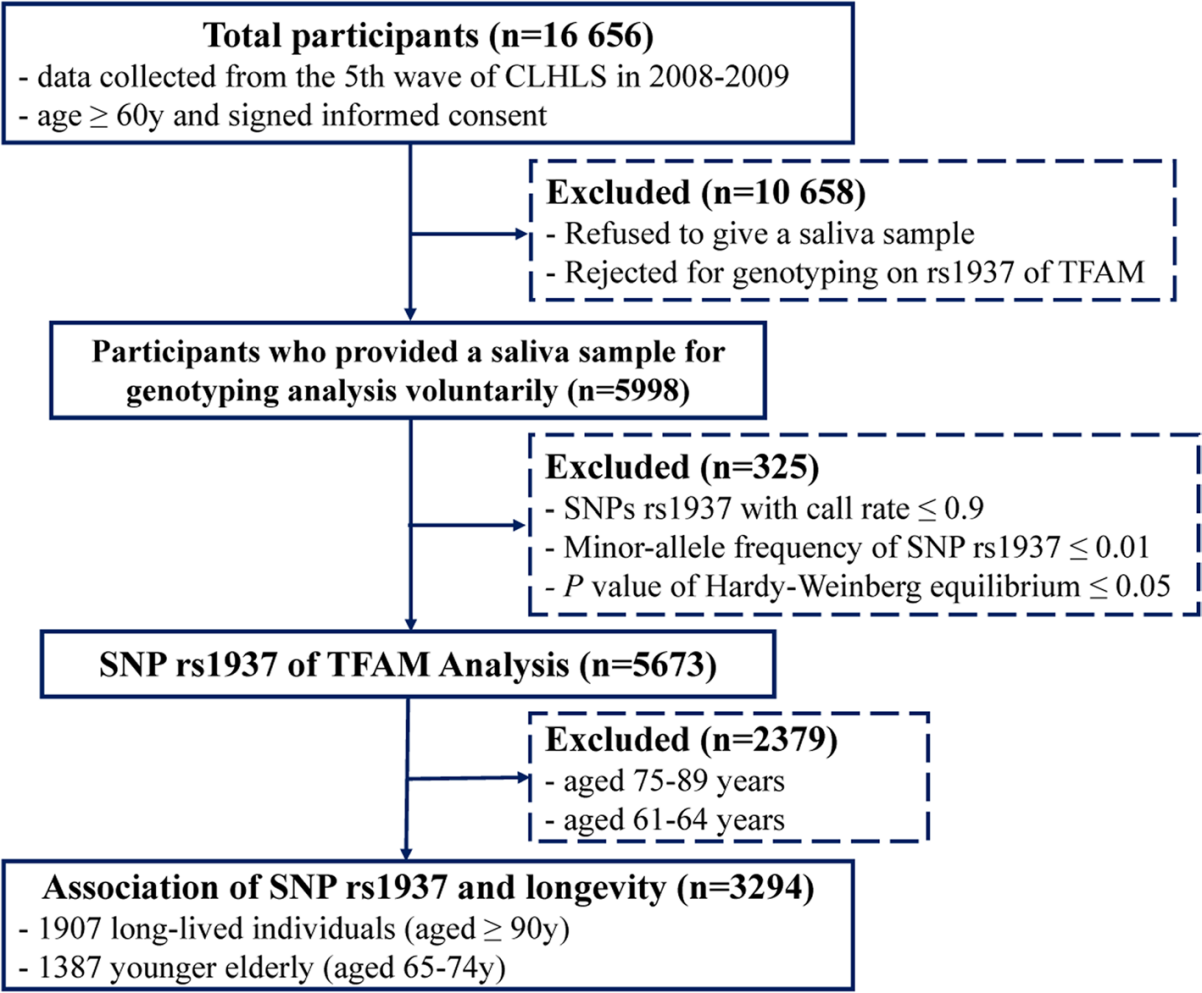
Flowchart of study participant

The genetic data of rs1937 on TFAM were drawn from the GWAS dataset of CLHLS. The participants whose SNP rs1937 with call rate ≤ 0.9, MAF ≤ 0.01, the inbreeding coefficient |F| > 0.1 or identity by descent (IBD) > 0.1 were excluded, 3294 individuals who provided a saliva sample and agreed to participate in genotyping assay were finally included in the analysis. The flowchart of the study participant is shown in Fig. 1.

### Statistical analysis

Mean and standard deviation (SD) (continuous variables) or numbers and percentages (categorical variables) were used to describe the research variables with normal distribution. And the median (interquartile range [*IQR*]) was used to describe the research variables with skewed distribution. Allele and genotype frequencies were estimated by Hardy-Weinberg equilibrium using the Chi-square test. Binary logistic regression was applied to calculate the odds ratio (OR) with 95% confidence interval (95% *CI*) for the association between the SNP rs1937 and longevity. Four sets of models were performed. Model 1 (basic model) was unadjusted for variables; Model 2 was adjusted for sex; Model 3 was further adjusted for body mass index, education, and status of living alone in model 2. Model 4 (the fully adjusted model) was adjusted for additional variables, including current smoking, current drinking as well as multimorbidity.

Stratified analyses were performed to calculate potential modification effects using the fully adjusted model. Furthermore, several sensitivity analyses were performed to test the reliability and robustness of our primary results by excluding the non-Han participants and dividing centenarians and nonagenarians into two longevity group. All statistical analyses were performed with R V.4.0.5 (R development Core Team, Vienna, Austria). All statistical data were deemed statistically significant with the two-sided test of *p* < 0.05.

## Results

### Study characteristics

The detailed information of the study participants is presented in Table 1. Of the 3294 participants, 1907 nonagenarians and centenarians were enrolled in the long-lived individuals group (median age: 94.0 years [*IQR*: 91.0–99.0]), of which 701 (36.8%) were men; 1387 young old were enrolled in the younger elderly group (median age: 70.0 years [*IQR*: 67.0–72.0]), of which 766 (55.2%) were men. In addition, the genotype frequencies in the long-lived individuals group were 69.3% GG, 27.5% GC, and 3.1% CC, the younger elderly group were 73.3% GG, 24.9% GC, and 1.7% CC, respectively. Compared to the younger elderly group, long-lived individuals were relatively more likely to be women, to be underweight, to be illiterate, and less likely to be current smoking, current drinking, and multimorbidity (all *p* < 0.001).

**Table 1:** Characteristics of study participants

Characteristics	Long-lived individuals	Younger elderly	*p* value
No. of participants	1907	1387	
Genotype			0.006
GG	1322 (69.3)	1017 (73.3)	
GC	525 (27.5)	346 (24.9)	
CC	60 (3.1)	24 (1.7)	
Age, median (*IQR*), years	94.0 (91.0–99.0)	70.0 (67.0–72.0)	< 0.001
Sex			< 0.001
Men	701 (36.8)	766 (55.2)	
Women	1206 (63.2)	621 (44.8)	
Body mass index (kg/m^2^)			< 0.001
Underweight	792 (41.5)	233 (16.8)	
Normal weight	948 (49.7)	791 (57.0)	
Overweight or obesity	167 (8.8)	363 (26.2)	
Education^a^			< 0.001
Illiteracy	1422 (74.7)	472 (34.1)	
Literacy	482 (25.3)	914 (65.9)	
Living alone			< 0.001
No	1601 (84.0)	1227 (88.5)	
Yes	306 (16.0)	160 (11.5)	
Current smoking			< 0.001
No	1640 (86.0)	986 (71.1)	
Yes	267 (14.0)	401(28.9)	
Current drinking			< 0.001
No	1581 (82.9)	1037 (74.8)	
Yes	326 (17.1)	350 (25.2)	
Multimorbidity			< 0.001
No	1537 (80.6)	967 (69.8)	
Yes	370 (19.4)	419 (30.2)	

*IQR* interquartile range

^a^Literacy was defined as receiving a formal education of more than one year, illiteracy was defined as receiving a formal education of less than one year

Values are numbers (percentages) unless stated otherwise

### Hardy-Weinberg equilibrium test

Allele frequencies of the major allele G among the long-lived individuals group were lower than the younger elderly group (83.1% vs 85.8%), and the minor allele C among the long-lived individuals group were higher than the younger elderly group (16.9% vs 14.2%), respectively (Table 2). The results showed that the expected and observed alleles distributions of rs1937 on TFAM were in good agreement with the Hardy-Weinberg equilibrium among both the long-lived individuals group (*χ*^*2*^ = 0.673, *p* = 0.412) and younger elderly group (*χ*^*2*^ = 0.609, *p* = 0.435) (Table 2). It indicated that participants in this study were from large groups and had good stability and representation of the elderly Chinese population.

**Table 2:** Hardy-Weinberg Equilibrium test and allele frequencies of rs1937

Allele	Long-lived individuals	Younger elderly
G (major allele)	3169 (83.1)	2380 (85.8)
C (minor allele)	645 (16.9)	394 (14.2)
*χ*^*2*^ value	0.673	0.609
*p* value	0.412	0.435

Values are numbers (percentages) unless stated otherwise

### Association between SNP rs1937 on TFAM and longevity

Table 3 presents SNP rs1937 on TFAM and their association with longevity. In the fully adjusted model (model 4), compared to younger elderly, long-lived individuals with “CC genotype” were more closely related to increased longevity than those with “GG genotype” (OR: 1.989, 95% *CI*, 1.160–3.411, *p* = 0.012); moreover, in the dominant model, long-lived individuals with “GC + CC genotype” were slightly associated with increased longevity than those with “GG genotype” (OR: 1.222, 95% *CI*, 1.025–1.457, *p* = 0.025). However, there were no significant associations between the “GC genotype” of rs1937 on TFAM and longevity (*p* > 0.05). Besides, model 4 had the highest prediction performance among the four models (AUC: 0.781, Nagelkerke R Square: 0.306) (Fig. S1).

**Table 3:** Association between rs1937 and longevity among the elderly Chinese population

rs1937	Model 1	Model 2	Model 3	Model 4
	OR (95%CI) *p* value	OR (95%*CI*) *p* value	OR (95%*CI*) *p* value	OR (95%*CI*) *p* value
GG	1.00 (Reference)	1.00 (Reference)	1.00 (Reference)	1.00 (Reference)
GC	1.167 (0.997–1.368) 0.056	2.122 (1.843–2.444) 0.037	1.148 (0.963–1.369) 0.124	1.169 (0.975–1.401) 0.091
CC	1.923 (1.190–3.109) 0.008	1.854 (1.139–3.018) 0.013	1.950 (1.136–3.348) 0.015	1.989 (1.160–3.411) 0.012
GC + CC	1.216 (1.043–1.418) 0.013	1.231 (1.053–1.440) 0.009	1.198 (1.008–1.425) 0.040	1.222 (1.025–1.457) 0.025

*OR* odds ratio, *CI* confidence interval

Model 1: unadjusted for variables. Model 2: adjusted for sex. Model 3: further adjusted for body mass index, education, and status of living alone in model 2. Model 4: further adjusted for current smoking, current drinking as well as multimorbidity in model 3

### Stratified and sensitivity analyses

In addition, we performed stratified analyses using the fully adjusted model (Table 4). We found that the associations between SNP rs1937 on TFAM and longevity were not significantly modified by sex, BMI, education, current smoking, current drinking as well as multimorbidity (all *p* for interaction > 0.05). However, the association between SNP rs1937 on TFAM and longevity was stronger among the participants who are not living alone (*p* for interaction < 0.001) (Table 4). Sensitivity analyses on the associations of rs1937 with longevity were broadly consistent when we excluded non-Han participants (Additional file 1: Table S2); and when nonagenarians and centenarians were divided into two longevity groups (Additional file 1: Table S3); and when controlled for the 5, 10-methylenetetrahydrofolate reductase (MTHFR) gene (rs1801131 and rs9651118) in the models, respectively (Additional file 1: Table S4).

**Table 4:** Stratified Analyses of Association between rs1937 and longevity among the elderly Chinese population

Subgroup	GG	GC	CC	*p* for interaction
	OR (95%*CI*)	OR (95%*CI*)	OR (95%*CI*)	
Sex				0.626
Men	1.00 (Reference)	1.088 (0.842–1.407)	1.657 (0.756–3.713)	
Women	1.00 (Reference)	1.263 (0.971–1.649)	2.529 (1.208–5.736)	
Body mass index (kg/m^2^)				0.879
Underweight	1.00 (Reference)	1.136 (0.803–1.619)	1.426 (0.568–4.132)	
Normal weight	1.00 (Reference)	1.179 (0.929–1.450)	1.886 (0.896–4.226)	
Overweight or obesity	1.00 (Reference)	1.055 (0.651–1.696)	3.199 (1.084–9.830)	
Education				0.623
Illiteracy	1.00 (Reference)	1.181 (0.923–1.517)	2652 (1.231–6.624)	
Literacy	1.00 (Reference)	1.133 (0.862–1.487)	1.634 (0.732–3.570)	
Living alone				< 0.001
No	1.00 (Reference)	1.098 (0.903–1.337)	3.130 (1.701–6.042)	
Yes	1.00 (Reference)	1.760 (1.062–2.978)	0.317 (0.095–1.033)	
Current smoking				0.244
No	1.00 (Reference)	1.247 (1.017–1.532)	2.343 (1.289–4.453)	
Yes	1.00 (Reference)	0.936 (0.623–1.399)	1.110 (0.324–3.793)	
Current drinking				0.488
No	1.00 (Reference)	1.103 (0.890–1.353)	2.149 (1.157–4.159)	
Yes	1.00 (Reference)	1.403 (0.936–2.107)	1.727 (0.622–5.133)	
Multimorbidity				0.132
No	1.00 (Reference)	1.131 (0.917–1.398)	1.441 (0.788–2.705)	
Yes	1.00 (Reference)	1.267 (0.887–1.811)	5.718 (1.880–21.737)	

*OR* odds ratio, *CI* confidence interval

Adjusted for sex, body mass index, education, and status of living alone, current smoking, current drinking as well as multimorbidity

## Discussion

In this population-based case-control study involving 3294 individuals, we found that the long-lived individuals (nonagenarians and centenarians) with the “CC genotype” of rs1937 in TFAM gene were more closely associated with promoting longevity than those with the “GG genotype” (OR: 1.989, 95% *CI*, 1.160–3.411). In the dominant model, the long-lived individuals with the “GC + CC genotype” of rs1937 were positively correlated with longevity (OR: 1.222, 95% *CI*, 1.025–1.457).

Although previous population-based research on the genetic associations of SNPs on TFAM with longevity are scarce, epidemiological evidence of a correlation between rs1937 on TFAM and neurodegeneration diseases [13, 19–21], including AD that has been well established. The findings of our study are in accordance with the results of several prior studies conducted in China, the United States, and European countries. These studies indicated that the rare “CC genotype” of rs1937 was positively associated with late-onset AD [19, 21] while the “GG genotype” was a risk factor for AD [10, 21]. And Catalina and colleagues [23] found a marginal association might exist for rs1937 with longevity. Similarly, we also found a positive association between the “CC genotype” of rs1937 and longevity among the Chinese long-lived population, which possibly may be a potential therapeutic target in the future.

Meanwhile, it has been proved that gender has an effect on longevity and the regulation mechanism of mitochondrial [27, 36–38]. Interestingly, in our study, participants with the “CC genotype” or “GC genotype” of rs1937 of TFAM were found to be no association with longevity between men and women (*p* for interaction = 0.626). It may be possible that the small population of centenarians and nonagenarians in this study caused this phenomenon. However, further researches are needed to prove the result because we cannot exclude the possibility that women long-lived individuals with the “CC genotype” of rs1937 may be related to longevity.

Although little is known about the genetic association on rs1937 of TFAM and longevity, the role of TFAM in the mtDNA repair and maintenance has been recently thoroughly reviewed. The mechanism of TFAM therefore will be mentioned here only briefly. Mitochondria play key roles in cellular energy supply and antimicrobial immune response and keep normal cellular functions [39–41] under physiological and pathological conditions. Previous researches have shown that mtDNA damage activates the complex DNA repair response to maintain genome integrity [42, 43], if the damage is not repaired in time or effectively enough, cellular senescence or apoptosis will be induced [44]. Clara and colleagues [45] have indicated that mitochondria are the candidate target for interventions to reduce the deleterious impact of senescence in ageing tissues. These findings thus hypothesized that TFAM might be associated with longevity.

In this case-control study, to our knowledge, it is the first attempt to explore the possible association between the SNP rs1937 of TFAM and longevity among Chinese nonagenarians and centenarians, which is of great significance to enrich the researches on the occurrence and development of longevity in Chinese elderly. It hence may not only enable researchers to further explore the mechanism of human longevity from the level of genetic polymorphisms, but also provide evidence support for gene diagnosis and therapeutic target for extending life spans in the future. Furthermore, like other genetic association studies on longevity, we have adjusted various potential confounders to optimize the results of the present study, including sex, body mass index, education, and status of living alone, current smoking, current drinking as well as multimorbidity.

There are several limitations and open questions in this study. First, potential selection bias may occur during the recruitment of the study population who provided a saliva sample voluntarily. Second, as in any observational study, unmeasured or residual confounding could be the foundation for positive associations and many causes of longevity are still unknown. Third, the genetic data in the current study was restricted to younger elderly aged 65–74 and long-lived individuals aged 90–112, therefore it may not be able to perform replication and validation studies. In addition, the limitations of the relatively small sample sizes and insufficient data for participants, especially for men centenarians, made it difficult to draw firm conclusions. Furthermore, our study did not check the results of genotyping on chips by another alternative, more reliable methods (such as Sanger sequencing, polymerase chain reaction - restriction fragment length polymorphism [PCR-RFLP], reverse transcription - polymerase chain reaction [RT-PCR]). And this might be one of the major limitations of the underlying associations of the TFAM rs1937 SNP with longevity. Additionally, the association of genetic polymorphisms and microenvironmental factors with longevity remains unknown, which may affect the results. Previous studies have shown that polygenes cannot independently affect longevity, but the human longevity is closely related to joint effects of various factors, such as diet, environmental factors, economic conditions, and individual psychological factors [46]. Therefore, it is difficult to rule out the potential influence of these external factors on life expectancy. Nonetheless, SNP-based tools may still facilitate the design and evaluation of diagnostic, preventive, or therapeutic support tools for longevity. Further well-designed studies in different population are required to illustrate the exact potential role of the TFAM rs1937 polymorphism in influencing mitochondrial function and hence longevity.

## Conclusions

In summary, the “CC genotype” of rs1937 polymorphism on TFAM is closely related to increased longevity among the Chinese old adults. Further studies are necessary to elucidate the role of rs1937 of TFAM in the biological processes of longevity.

## Supplementary Information

**Additional file 1: Table S1.** Characteristics of included participants compared to results of excluding non-Han participants. **Table S2.** Association between rs1937 and longevity among Han-participants. **Table S3.** Association between rs1937 and longevity among centenarians and nonagenarians. **Table S4.** Association between rs1937 and longevity after adjusting for the rs1801131 and rs9651118 of the MTHFR gene. **Figure S1.** ROC curve of the four sets of models in Table 3.

## Data Availability

The CLHLS questionnaires are openly available at https://sites.duke.edu/centerforaging/programs/chinese-longitudinal-healthy-longevity-survey-clhls/. The genetic data used in this study are available from the corresponding author upon reasonable request.

## Abbreviations

TFAM: Mitochondrial transcription factor A
SNP: Single nucleotide polymorphism
CLHLS: Chinese Longitudinal Healthy Longevity Survey
mtDNA: Mitochondrial DNA
ROS: Reactive oxygen species
ATP: Adenosine triphosphate
AD: Alzheimer’s disease
BMI: Body mass index
GWAS: Genome-wide association study
MAF: Minor-allele frequency
1KGP: The 1000 Genomes Project
IBD: Identity by descent
SD: Standard deviation
IQR: Interquartile range
OR: Odds ratio
CI: Confidence interval
PCR-RFLP: Polymerase chain reaction - restriction fragment length polymorphism
RT-PCR: Reverse transcription - polymerase chain reaction
