# Supplementary material for: Association between single nucleotide polymorphism of rs1937 in TFAM gene and longevity among the elderly Chinese population: based on the CLHLS study
Source: BMC Geriatr. 2022 Jan 3;22:16. doi: 10.1186/s12877-021-02655-3 (PMC8722189; doi:10.1186/s12877-021-02655-3)
Supplement: Supplementary file 1 — Additional file 1: Table S1. Characteristics of included participants compared to results of excluding non-Han participants. Table S2. Association between rs1937 and longevity among Han-participants. Table S3. Association between rs1937 and longevity among centenarians and nonagenarians. Table S4. Association between rs1937 and longevity after adjusting for the rs1801131 and rs9651118 of the MTHFR gene. Figure S1. ROC curve of the four sets of models in Table 3. [file 12877_2021_2655_MOESM1_ESM.docx]

**Association between single nucleotide polymorphism of rs1937 in TFAM gene and longevity among the elderly Chinese population: based on the CLHLS study**

**Supplementary Information**

Additional file 1:

**Table S1 Characteristics of included participants compared to results of excluding non-Han participants**

| Characteristics | Participants included  (n =3294) | Han-Participants  (n =3051) |
| --- | --- | --- |
| Genotype |  |  |
| GG | 2339 (71.0) | 2165 (71.0) |
| GC | 871 (26.4) | 804 (26.4) |
| CC | 84 (2.6) | 82 (2.7) |
| Age, median (*IQR*), years |  |  |
| Younger elderly | 70.0 (67.0 - 72.0) | 70.0 (67.0 - 72.0) |
| Long-lived individuals | 94.0 (91.0 - 99.0) | 94.0 (91.0 - 99.0) |
| Sex |  |  |
| Men | 1467 (44.5) | 1364 (44.7) |
| Women | 1827 (55.5) | 1687 (55.3) |
| Body mass index (kg/m^2^) |  |  |
| Underweight | 1025 (31.1) | 928 (30.4) |
| Normal weight | 1739 (52.8) | 1619 (53.1) |
| Overweight or obesity | 530 (16.1) | 504 (16.5) |
| Education^*^ |  |  |
| Illiteracy | 1894 (57.6) | 1746 (57.3) |
| Literacy | 1395 (42.4) | 1300 (42.7) |
| Living alone |  |  |
| No | 2828 (85.9) | 2617 (85.8) |
| Yes | 466 (14.1) | 434 (14.2) |
| Current smoking |  |  |
| No | 2626 (79.7) | 2417 (79.2) |
| Yes | 668 (20.3) | 634 (20.8) |
| Current drinking |  |  |
| No | 2618 (79.5) | 2459 (78.8) |
| Yes | 676 (20.5) | 663 (21.2) |
| Multimorbidity |  |  |
| No | 2504 (76.0) | 2419 (79.3) |
| Yes | 789 (24.0) | 632 (20.7) |

IQR: interquartile range;

^*^Literacy was defined as receiving a formal education of more than one year, illiteracy was defined as receiving a formal education of less than one year.

Values are numbers (percentages) unless stated otherwise.

**Table S2. Association between rs1937 and longevity among Han-participants**

| rs1937 | Model 1  OR (95%CI) *p* value | Model 2  OR (95%*CI*) *p* value | Model 3  OR (95%*CI*) *p* value | Model 4  OR (95%*CI*) *p* value |
| --- | --- | --- | --- | --- |
| GG | 1.00 (Reference) | 1.00 (Reference) | 1.00 (Reference) | 1.00 (Reference) |
| GC | 1.168 (0.991 - 1.378) 0.064 | 1.187 (1.004 - 1.404) 0.045 | 1.140 (0.947 - 1.374) 0.168 | 1.168 (0.967 - 1.412) 0.108 |
| CC | 2.002 (1.245 - 3.328) 0.005 | 1.943 (1.200 - 3.251) 0.009 | 2.056 (1.199 - 3.633) 0.011 | 2.112 (1.233 - 3.727) 0.008 |
| GC + CC | 1.224 (1.044 - 1.436) 0.013 | 1.238 (1.053 - 1.457) 0.010 | 1.198 (1.001 - 1.436) 0.050 | 1.230 (1.025 - 1.478) 0.027 |

OR: odds ratio; CI: confidence interval;

Model 1: unadjusted for variables; Model 2: adjusted for sex; Model 3: further adjusted for body mass index, education, and status of living alone in model 2; Model 4: further adjusted for current smoking, current drinking as well as multimorbidity in model 3.

**Table S3 Association between rs1937 and longevity among centenarians and nonagenarians**

| Genotype | **Centenarians**  Model 1^a^  OR (95%CI) *p* value | Model 2^b^  OR (95%*CI*) *p* value | **Nonagenarians**  Model 1^a^  OR (95%*CI*) *p* value | Model 2^b^  OR (95%*CI*) *p* value |
| --- | --- | --- | --- | --- |
| GG | 1.00 (Reference) | 1.00 (Reference) | 1.00 (Reference) | 1.00 (Reference) |
| GC | 1.593 (1.268-1.996) ＜0.001 | 1.678 (1.268-2.221) ＜0.001 | 1.043 (0.879-1.238) 0.629 | 1.050 (0.867-1.273) 0.616 |
| CC | 1.984 (0.989-3.835) 0.046 | 2.290 (0.962-5.420) 0.060 | 1.905 (1.166-3.192) 0.012 | 2.019 (1.165-3.575) 0.014 |
| GC + CC | 1.618 (1.296-2.017) ＜0.001 | 1.714 (1.305-2.254) ＜0.001 | 1.099 (0.932-1.297) 0.263 | 1.112 (0.923-1.329) 0.264 |

Take the centenarians (aged 100 years and older) and nonagenarians (aged 90-99 years) as the longevity group, and the younger elderly (aged 65-74 years) as the control group; OR: odds ratio; CI: confidence interval;

^a^ Model 1: unadjusted for variables;

^b^ Model 2: adjusted for sex, body mass index, education, and status of living alone, current smoking, current drinking as well as multimorbidity.

**Table S4 Association between rs1937 and longevity** **after adjusting for the rs1801131 and rs9651118 of the MTHFR**

| rs1937 | Model 1  OR (95%CI) *p* value | Model 2  OR (95%*CI*) *p* value |
| --- | --- | --- |
| GG | 1.00 (Reference) | 1.00 (Reference) |
| GC | 1.196 (0.975 - 1.402) 0.092 | 1.168 (0.974 - 1.401) 0.094 |
| CC | 1.967 (1.159 - 3.433) 0.014 | 1.951 (1.148 - 3.407) 0.016 |
| GC + CC | 1.221 (1.024 - 1.456) 0.027 | 1.218 (1.022 - 1.453) 0.028 |

MTHFR: 5, 10-methylenetetrahydrofolate reductase; OR: odds ratio; CI: confidence interval;

Model 1: adjusted for sex, body mass index, education, and status of living alone, current smoking, current drinking, multimorbidity as well as rs1801131;

Model 2: further adjusted for the rs1801131 and rs9651118 of the MTHFR gene in model 1.


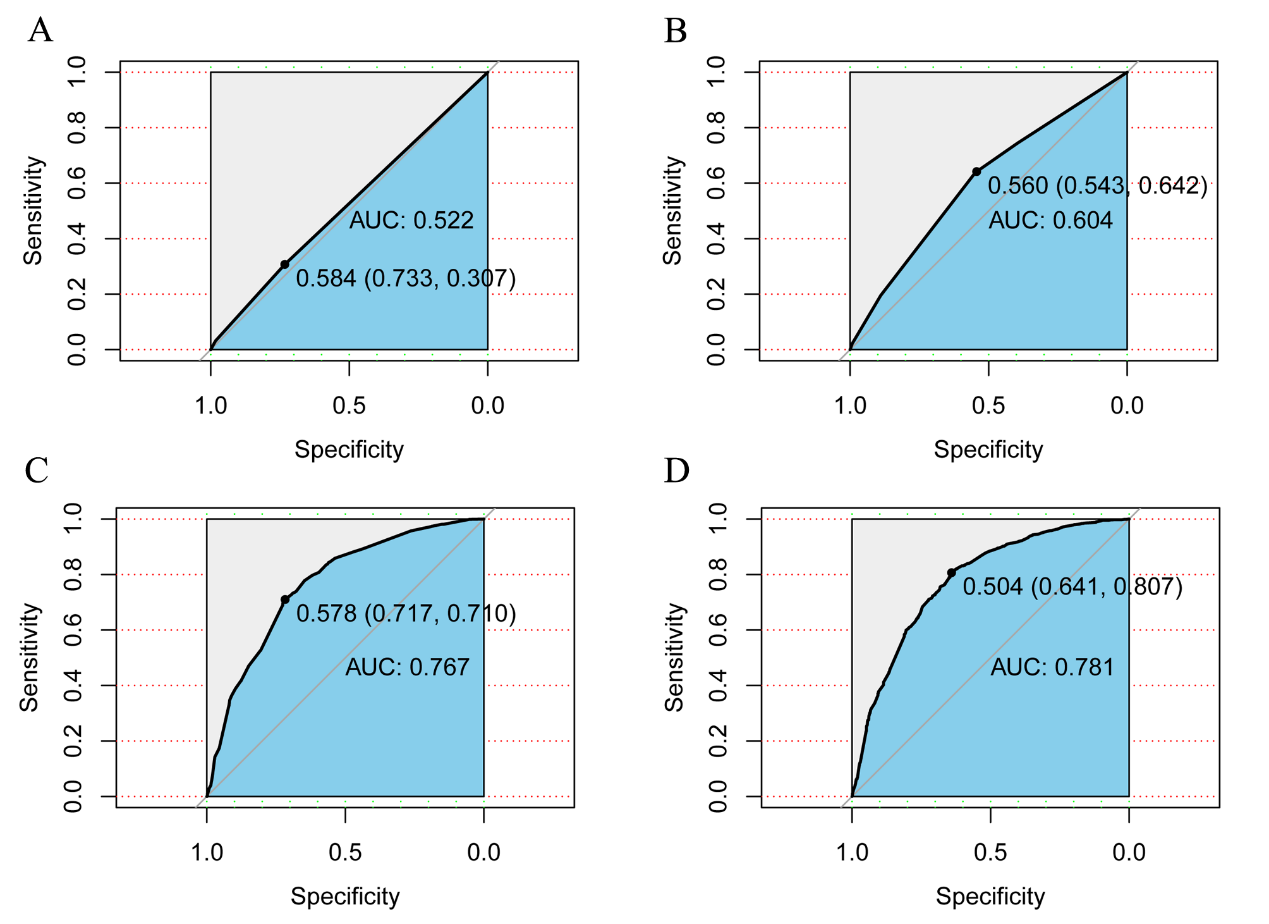


**Fig.S1**

ROC curve of the four sets of models in table 3. **a** Model 1: unadjusted for variables. **b** Model 2: adjusted for sex. **c** Model 3: further adjusted for body mass index, education, and status of living alone in model 2. **d** Model 4: further adjusted for current smoking, current drinking as well as multimorbidity in model 3.
